# Occupational Therapy in HomEcare Re-ablement Services (OTHERS): results of a feasibility randomised controlled trial

**DOI:** 10.1136/bmjopen-2016-011868

**Published:** 2016-08-15

**Authors:** Phillip J Whitehead, Marion F Walker, Ruth H Parry, Zaid Latif, Ian D McGeorge, Avril E R Drummond

**Affiliations:** 1 School of Medicine, University of Nottingham, Nottingham, UK; 2 School of Health Sciences, University of Nottingham, Nottingham, UK; 3 Nottingham City Council, Nottingham, UK; 4 Absoluteco, Nottingham, UK

**Keywords:** Occupational Therapy, Activities of Daily Living, Homecare Re-ablement, Social Care, Prevention

## Abstract

**Objectives:**

The objective of this study was to test the feasibility of conducting a randomised controlled trial (RCT) of an intervention targeted at activities of daily living (ADL), delivered by an occupational therapist, in homecare reablement.

**Design:**

Feasibility parallel group RCT.

**Setting:**

Single-site local authority homecare reablement service.

**Participants:**

People referred for homecare reablement with ability to consent. Exclusion criteria were as follows: inability to speak English, receiving other community therapy services, needing two or more to assist transfer and receiving end-of-life care.

**Control:**

‘Usual care’ was 6 weeks of homecare reablement delivered by social care workers (no routine health professional input).

**Intervention:**

A targeted ADL programme, delivered by an occupational therapist incorporating goal setting, teaching/practising techniques, equipment/adaptations and provision of advice/support. This was in addition to usual care.

**Outcome measures:**

Aspects of feasibility including eligibility, recruitment, intervention delivery, attrition and suitability and sensitivity of outcome measures. Participant outcomes were personal and extended ADL, quality of life, falls and use of health and social care services.

**Results:**

30 participants were recruited, 15 to each arm, which was 60% of those eligible. Data from 22 (73%) were analysed at 6 months. Of the 15 participants, 13 (86%) received the intervention and were able to set one or more ADL goals. There were improvements from baseline in both groups, although overall improvements were greater in the occupational therapy (OT) intervention group. The biggest threat to feasibility was a change in service configuration during the trial, involving additional occupational therapy input, affecting usual care and recruitment.

**Conclusions:**

Despite the service reconfiguration, it was feasible to recruit and retain participants, deliver the intervention and collect outcome data that were responsive to change. The choice of primary outcome measure remains unclear. A further powered study is feasible and warranted; however, the design will require careful consideration because of ongoing national changes in service configurations.

**Trial registration number:**

ISRCTN21710246; Results.

**Strengths and limitations of this study**

- This is the first feasibility randomised controlled trial (RCT) of occupational therapy in homecare reablement and one of a few RCTs in a social care setting.
- The study was conducted at one site with one occupational therapist delivering the intervention. Further research is needed to ascertain whether intervention delivery could be standardised across sites.
- The choice of primary outcome measure remains unclear.
- There were trial difficulties because of changes in routine care in parallel with national changes in reablement services.

## Introduction

Reablement services are currently high on the policy agenda.^1^ The Care Act 2014 has placed a statutory duty on local authorities in England to provide services that prevent or delay the need for other health and social care services, which may involve maximising independent living. Reablement is identified within The Care Act statutory guidance as an example of prevention^2^ and has been identified as one of the ‘top-ten’ prevention services for older adults.^3^ Traditionally, homecare services have involved paid care workers completing activities ‘for’ the person.^4^ In contrast, Homecare Re-ablement services aim to assist the person to maximise their ability to carry out activities independently with the aim of reducing the amount of paid care worker input required in the long term. Internationally, such services may be referred to as ‘restorative homecare’.^5^ ^6^ In the UK, Homecare Re-ablement Services are usually provided for up to 6 weeks after which time an assessment is made about the person's need for ongoing homecare.^7^ Some services may accept referrals for people being discharged from hospital, others will accept people already living in the community and some may accept referrals from both. Although studies have suggested that there are reductions in the amount of homecare provided following reablement in comparison to traditional homecare,^5^ ^7–11^ there are outstanding questions about the optimum model of service delivery: one such aspect is occupational therapy provision.^12^

There may be similarities between reablement and other rehabilitation services, and these terms are often used interchangeably;^2^ however, a feature of reablement services in the UK is that are commonly embedded within social care. The National Audit for Intermediate Care defines reablement as being predominantly delivered by social care professionals;^13^ these are often former homecare workers who are urged to ‘stand-back’ and encourage the user to carry out tasks independently wherever possible.^14^ Homecare Re-ablement Services are different to other community rehabilitation services, such as home-based intermediate care, which tend to have higher staffing ratios of health professionals including nurses and therapists.^13^ However, as reablement services have become more widely implemented, there has been an apparent increase in therapy input, notably an increase in those that are therapy-led from 9% in 2013 to 32% in 2014.^15^ Occupational therapists are argued to have a particularly important role to play in delivering successful reablement outcomes^16^ as services aim to support individuals to manage daily living tasks independently; this is a core aspect of occupational therapy practice.^17^ Furthermore, occupational therapists are the only allied health profession to be employed within social care services in significant numbers^18^ and thus are already working as social care professionals.

There are several ways in which occupational therapists might be involved in Homecare Re-ablement Services, including providing training to reablement workers, carrying out reviews of user progress, becoming involved in particular cases in an advisory capacity or working as core team members.^19^ The latter often involves working directly with service users delivering case-by-case programmes based on collaborative goal setting. Anecdotally, it is known that there are widespread differences between local authorities in terms of the type and extent of occupational therapy input into homecare reablement services. A systematic review of interventions to reduce dependency in activities of daily living (ADL) in homecare services was carried out as a precursor to this study.^20^ The review reported that occupational therapists were involved in 7 of the 13 of the included interventions, with the type of input varying. The majority of interventions showed small (but not statistically significant) improvements in ADL ability; however, it was not possible to determine whether those interventions involving occupational therapists led to better outcomes than those not involving them.

The Social Care Institute for Excellence stated that comparing “the effectiveness and cost-effectiveness of [re-ablement] services that employ occupational therapists as core team members with those that do not” was an important priority for further research.^19^ Thus, the aim of this study was to ascertain the feasibility of conducting a definitive, appropriately powered randomised controlled trial (RCT) of an occupational therapy-delivered intervention targeting ADL for people using homecare reablement services.

## Method

### Design

Single-centre feasibility RCT. Participants were individually randomised to parallel groups at a ratio of 1:1 intervention to control. The trial was registered on the current controlled trials register, ISRCTN21710246. The protocol was published prospectively.^21^

### Setting

The setting was a local authority homecare reablement service in England. The service accepted referrals from any adult aged over 18 years, with a need for homecare support with the exception of those with a diagnosis of dementia who already had a specialist dementia homecare service within the area. The service accepted referrals for people being discharged from hospital and those who were living within the community. People leaving hospital with new or increased difficulties with ADL would be particularly likely to be referred to the service. It was divided into six geographical subteams and the RCT recruited from three of the subteams, which did not have routine input from an occupational therapist at the time.

### Participants

All users of the service within the selected subteams were screened for eligibility. Inclusion criterion was the ability to provide informed written consent. Exclusion criteria were inability to speak English, on an end-of-life care pathway, requiring assistance from two or more to transfer or receiving input from a community rehabilitation team.

### Control

Those randomised to the control group received usual routine care provided by the homecare reablement service: a period of homecare reablement provided by reablement workers (social care workers) under the direction of a reablement care team leader (social care team manager), with a maximum target of 6-week duration. However, participants could remain in the service longer, particularly if they needed an ongoing care package and there were delays in providing this. The control group did not receive any routine input from qualified health professionals. Participants received visits from social care workers to assist them with daily living tasks, and there was an intention to reduce the amount of assistance over the 6 weeks wherever possible. If participants in the control group were identified as requiring specific occupational therapy input, they were referred to the mainstream community occupational therapy team (waiting time exceeding the 6-week reablement period). Referrals to occupational therapy were not made routinely.

### Intervention

Those randomised to the intervention group received all routine homecare reablement services and, in addition, received an enhanced programme targeted at ADL, delivered by an occupational therapist in their home. The aim of the enhanced programme was to maximise independence in ADL activities including, but not limited to, washing, dressing, bathing and showering, feeding, indoor mobility, transfers, stair mobility, toileting, meal preparation and kitchen activities, outdoor mobility and community access. The programme was agreed with the participant and individually tailored to their needs. It included the following: goal setting using the TARGET;^22^ practising activities, and/or a graded process of re-learning and building the skills to manage ADL independently; equipment provision and environmental or activity modification; and case management involving advice to the person and their support network. Weekly reviews were completed by the occupational therapist alongside liaison with other members of the team and other services as appropriate.

The intervention was based on occupational therapy principles and practices and the occupational therapy process,^23^ ^24^ the findings of a systematic review^20^ and interviews which were carried out with occupational therapists and reablement service users prior to this study. It was delivered by one occupational therapist (PW) who combined medical knowledge of prognosis with assessment of functional ability in order to select an appropriate approach for the reablement episode (eg, a compensatory or a biomechanical approach). Provision of community equipment and/or minor adaptations (such as grab rails, half-steps or threshold removal or replacements) formed a core component and were prescribed by the occupational therapist and provided by the Community Equipment Service for the local area. These were usually delivered within 1 week of prescription and were in the participant's home before the reablement service ended. The occupational therapy programme continued for the duration of the reablement episode and ceased when the participant was discharged from the reablement service. The aim of the programme was to use the occupational therapist's core skills in activity analysis and ADL in order to maximise independence in ADL where possible.

### Outcomes

The primary outcome was a composite measure to determine the feasibility of conducting an appropriately powered trial. The composite measure included an assessment of recruitment, retention and the viability of delivering the intervention. Key aspects to be addressed were as follows: whether the eligibility criteria were realistic, whether service users were willing to be randomised, the dropout rate, the content and scheduling of the occupational therapy treatment visits, the most suitable primary outcome measure for the definitive trial and the feasibility of the cost and resource use data collection. These data were collected from the screening and recruitment log, the intervention log and analysis of the completeness of the participant outcome data.

The participant outcomes to be assessed were as follows: personal and extended ADL, health-related and social care-related quality of life at 2 weeks, 3 months and 6 months post reablement. The measures were Barthel Index (BI),^25^ Nottingham Extended Activities of Daily Living (NEADL),^26^ Short-Form 36 (SF-36) Physical Component Summary (PCS) and Mental Component Summary (MCS),^27^ Adult Social Care Outcomes Toolkit (ASCOT),^28^ EuroQol EQ-5D-3L.^29^ As this study was based within social care services, measures of health-related and social care-related quality of life were included. The ASCOT is designed to capture the effects of social care interventions including domains such as personal cleanliness, comfort, safety, control and dignity.^28^ It also incorporates questions about the feelings associated with having assistance with particular activities, which is different to health-related quality of life constructs. Information was also collected on the number of homecare hours, falls, admissions (to acute and residential services) and use of health and community services.

The initial intention was to also include a measure of carer strain as detailed in the protocol.^21^ However, this would have required an additional consent process for carer participants and would have had to be collected separately to the service user data. It was therefore decided for pragmatic reasons to focus on collecting data on service user participants and on the feasibility of the intervention; thus, carers were not recruited.

### Randomisation and blinding

Participants were enrolled into the study by PW. Baseline assessments were completed prior to randomisation. Participants were randomised using web-based software developed by Nottingham Clinical Trials Unit (NCTU), which was administered by PW. Participants were individually randomised in random varying block sizes at a ratio of 1:1 (intervention to control). Only the NCTU had access to the allocation sequence. It was not possible to blind participants or staff due to the nature of the intervention. Outcome data were collected face to face by an assessor who was blind to treatment allocation and administered the questionnaires in the participant's home. Data were entered into a database by the same assessor. Baseline and outcome assessors received training in administering the measures.

### Sample size and analyses

As a feasibility study, no formal sample size calculation was required. The aim was to recruit ∼50 participants in order to inform a sample size calculation for a definitive RCT. Descriptive statistics were used for the feasibility and participant outcomes. The data for some measures were not normally distributed; thus, measures are presented using medians and interquartile ranges and change from baseline calculations. Between-group differences and their CIs were calculated, in order to assess the suitability and sensitivity of outcome measures and to estimate the treatment effects for evaluation in a powered study. p Values were not presented as this is not appropriate for feasibility studies.

## Results

### Recruitment, participant flow and service change

The trial opened for recruitment on 1 April 2014 and closed on 30 November 2014. The final follow-up visit was completed on 21 July 2015. Recruitment was based within three geographical subteams which varied over the course of the study due to operational issues within the service. An unanticipated issue which affected the recruitment rate was the introduction of new occupational therapists into the reablement service during the course of the study. Midway through the trial recruitment period, additional occupational therapists were employed to work within the service. However, the new occupational therapists had insufficient capacity to work with every service user and were allocated to particular geographical subteams within the authority. Therefore, the study continued within two geographical subteams where the additional occupational therapists were not employed (this was later reduced to one).

**Figure 1:**
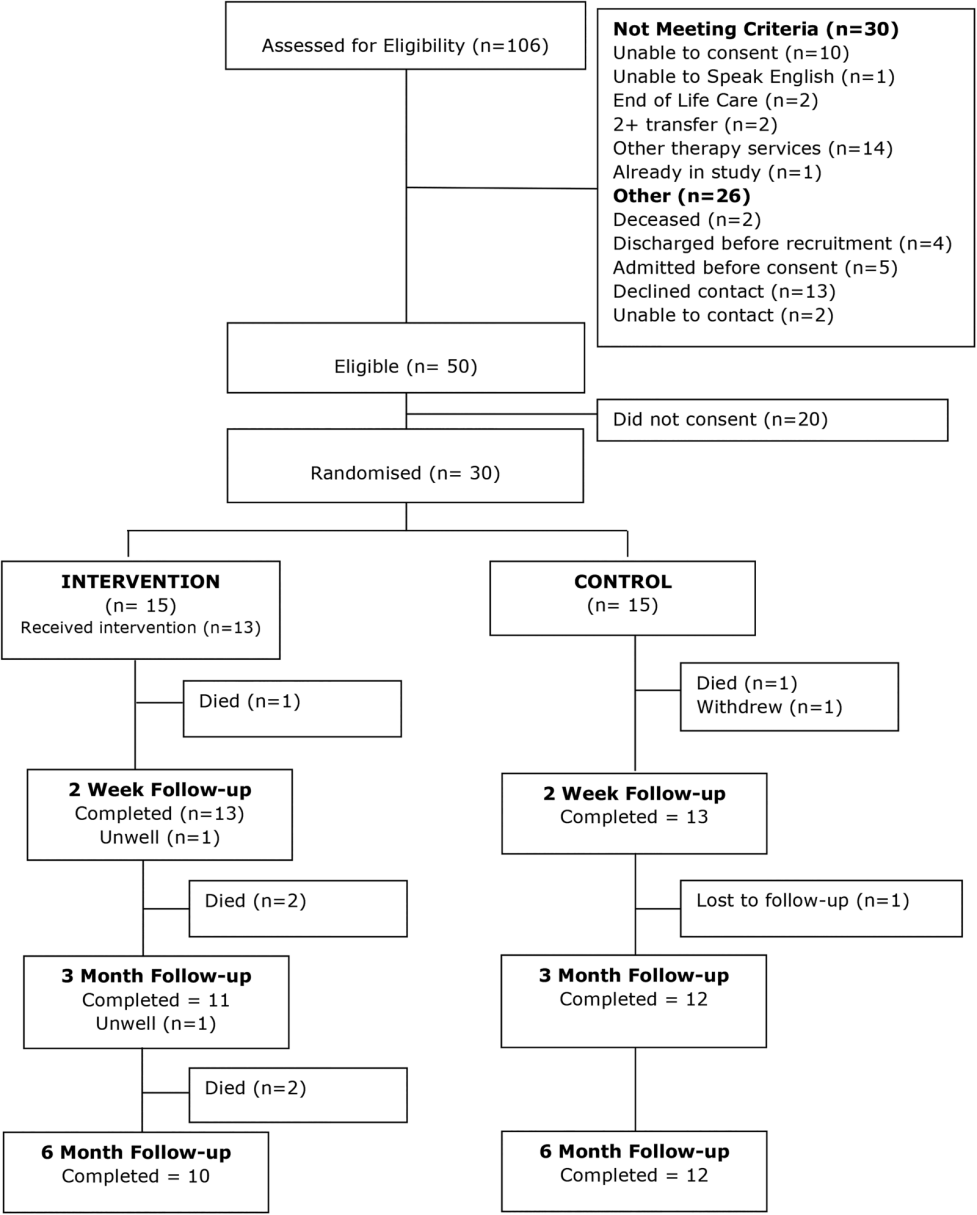
Flow of participants.

Figure 1 shows the recruitment figures and the flow of participants through the study. In total, 106 people were screened for eligibility. Of them, 30 were excluded because they did not meet the criteria, and 26 could not be approached for other reasons. The principal reason for exclusion was being in receipt of other community rehabilitation services (eg, the community stroke team) (n=14) and being unable to consent (n=10). A total of 50 people met the criteria and were approached; of them, 30 provided informed consent and were randomised, 15 to each arm. Figure 1 also shows attrition: 26 participants were followed up at 2 weeks, 23 at 3 months and 22 at 6 months. The main reason for attrition was death; six participants died. Of these, in the occupational therapy intervention group, two participants were admitted to hospital within a week of randomisation; both subsequently died in hospital and did not receive any intervention.

### Baseline data

The demographic characteristics and medical details of the participants are shown in table 1. The mean age reflects that, although the service was available to all adults of all ages, users of the service were predominantly older adults. There was a preponderance of men in the control group compared to the occupational therapy (OT) intervention group. There were also more people with primary medical category as ‘neurological conditions’ in the control group compared with ‘musculoskeletal conditions’ in the OT intervention group. These were broad categories which the local authority used; however, they are not mutually exclusive, and most participants had multiple morbidities. The groups were well matched on other variables. Table 2 shows the details of the baseline measures. The median in the OT intervention group was lower on all baseline measures than the control group. The exception was the mini-mental state examination (MMSE), which is used as a baseline descriptor only.

**Table 1:** Participant demographic and medical details

	OT intervention (n=15)	Control (n=15)
Gender		
Male	4 (27%)	9 (60%)
Age		
Mean (SD)	82.93 (9.02)	81.93 (12.96)
Lives alone		
Yes	9 (60%)	11 (73%)
Ethnicity		
White British	12 (80%)	14 (93%)
Other	3 (20%)	1 (7%)
Property ownership		
Owner occupier	10 (66%)	12 (80%)
Local authority	3 (20%)	2 (13%)
Housing association	1 (7%)	1 (7%)
Privately rented	1 (7%)	0 (0%)
Employment status		
Retired	15 (100%)	13 (87%)
Unemployed	0 (0%)	2 (13%)
Hospital/community referral		
Hospital	12 (80%)	10 (67%)
Community	3 (20%)	5 (33%)
Informal carer		
No	4 (27%)	5 (33%)
Within household	6 (40%)	4 (27%)
External to household	5 (33%)	6 (40%)
Primary medical category		
Neurological	0 (0%)	5 (33%)
Musculoskeletal	11 (73%)	5 (33%)
Frailty	1 (7%)	3 (20%)
Mental health	0 (0%)	2 (14%)
Other	3 (20%)	0 (0%)

**Table 2:** Participant baseline measures

Measure	OT intervention median (IQR) (n=15)	Control median (IQR) (n=15)
BI	16 (14–17)	17 (16–18)
NEADL	19 (12–28)	20 (16–28)
EQ5D	0.27 (0.08–0.59)	0.59 (0.08–0.64)
ASCOT	0.72 (0.55–0.84)	0.77 (0.56–0.84)
SF-36 PCS	27.01 (20.28–33.02)	29.33 (20.4–39)
SF-36 MCS	48.50 (33.98–54.03)	52.36 (45.23–55.26)
MMSE*	27 (24–28)	26 (23–28)

For all measures higher scores indicate better outcomes.

BI, Barthel index, scale: 0–20; NEADL, Nottingham Extended Activities of Daily Living, scale: 0–66; EQ5D, EQ-5D-3L, scale: −0.11 to 1; ASCOT, Adult Social Care Outcomes Toolkit, scale: 0–1; SF-36 PCS, Short-Form 36 Physical Component Summary, scale: 0–100; SF-36 MCS, Short-Form 36 Mental Component Summary, scale: 0–100; MMSE, mini-mental state examination, scale: 0–30.

*MMSE was completed with 14 intervention participants and 13 control participants. One declined to complete it, one could not complete in the allocated time and one was unable due to speech and language impairment.

### Participant outcomes

The medians on all measures increased from baseline at all time points in the OT intervention group, compared to 9/18 in the control group. However, it is important to note that the groups were different at baseline, both in relation to their scores on the measures and the preponderance of men in the control group. Change from baseline scores were therefore calculated for each outcome, and a linear regression was carried out in order to adjust for the between-group difference in gender and provide the most accurate estimation of the treatment effect for detection in a powered study. The adjusted results are presented in table 3. The direction of the change favours the OT intervention group in 15/18 measures and time points; however, CIs were wide reflecting the small sample size. Positive trends were particularly evident for social care-related quality of life and mental well-being, which were consistent across all time points. Data on self-reported falls are presented in table 4; there were fewer falls in the OT intervention group in terms of the number of participants who reported a fall and the mean number of falls reported.

**Table 3:** Participant outcomes—change from baseline adjusted for gender

	Change from baseline OT—control (SE) 95% CI		
Measure	2 weeks (n=26)	3 months (n=23)	6 months (n=22)
BI	0.7 (1.08) −1.52 to 2.93	−0.13 (1.33) −2.91 to 2.65	0.28 (1.12) −2.06 to 2.61
NEADL	−2.43 (4.59) −11.92 to 7.07	3.72 (4.58) −5.83 to 13.27	1.58 (5.28) −9.47 to 12.64
EQ5D	0.06 (0.17) −0.30 to 0.42	−0.03 (0.15) −0.35 to 0.28	0.23 (0.22) −0.23 to 0.69
ASCOT	0.07 (0.08) −0.09 to 0.23	0.06 (0.11) −0.18 to 0.30	0.04 (0.10) −0.17 to 0.25
SF-36 PCS	3.63 (3.38) −3.36 to 10.64	1.52 (4.75) −8.43 to 11.47	0.09 (5.33) −11.06 to 11.24
SF-36 MCS	6.60 (4.53) −2.80 to 16.00	7.84 (3.17) 1.17 to 14.51*	3.39 (4.90) −6.88 to 13.66

BI, Barthel index, scale: 0–20; NEADL, Nottingham Extended Activities of Daily Living, scale: 0–66; EQ5D, EQ-5D-3L, scale: −0.11 to 1; ASCOT, Adult Social Care Outcomes Toolkit, scale: 0–1; SF-36 PCS, Short-Form 36 Physical Component Summary, scale: 0–100; SF-36 MCS, Short-Form 36 Mental Component Summary, scale: 0–100; MMSE, mini-mental state examination, scale: 0–30.

*One outlier was removed from the analysis of the MCS at 3 months who had an extreme change score of −35.81, which caused a skew of the data.

**Table 4:** Number of reported participant falls

	Group	2 Weeks	3 Months	6 Months
Participants with one or more falls	OT	2/13 (15%)	2/11 (18%)	2/10 (20%)
	Control	4/13 (31%)	3/12 (25%)	6/12 (50%)
Number of falls per participant (with a fall), mean (SD)	OT	1 (0)	1 (0)	1 (0)
	Control	2.75 (1.70)	1.67 (1.15)	1.5 (1.22)

Data show the falls reported during each separate time period.

### Service use outcomes

Table 5 shows the information collected on the use of health and social care services during follow-up (resource use), presented as the number of participants who used each service during the time period. This refers to the use of services after the reablement service had ended. Information was also collected on the amount of time used per service, for the purposes of calculation of resource use. It was possible to collect this information, but as the numbers were small, these data are not presented here.

**Table 5:** Use of health and social care services during follow-up

Outcome	Group	2 Weeks	3 Months	6 Months
Participants with homecare package*	OT	6/13 (46%)	2/11 (18%)	6/10 (60%)
	Control	8/13 (62%)	6/12 (50%)	6/12 (50%)
Hospital admission	OT	1/13 (8%)	3/11 (27%)	4/10 (40%)
	Control	0/13 (0%)	2/12 (17%)	2/12 (17%)
Residential/nursing admission	OT	0/13 (0%)	1/11 (9%)	0/10 (0%)
	Control	1/13 (8%)	0/12 (0%)	0/12 (0%)
Outpatient health services (including GP)†	OT	4/13 (31%)	6/11 (55%)	6/10 (60%)
	Control	7/13 (54%)	6/12 (50%)	12/12 (100%)
Occupational therapist	OT	0/13 (0%)	0/11 (0%)	0/10 (0%)
	Control	1/13 (8%)	2/12 (17%)	2/12 (17%)
Health professional at home (other than OT)‡	OT	8/13 (62%)	7/11 (64%)	6/10 (60%)
	Control	5/13 (38%)	9/12 (75%)	6/12 (50%)
Meals at home	OT	0/13 (0%)	0/11 (0%)	1/10 (10%)
	Control	2/13 (15%)	2/12 (17%)	3/12 (25%)
Day centre§	OT	1/13 (8%)	0/11 (0%)	1/10 (10%)
	Control	0/13 (0%)	1/12 (8%)	1/12 (8%)

*One or more visit per week from a paid care worker to assist with an activity of daily living within the home environment.

†Any visit to a health professional or health service that did not involve an admission or overnight stay.

‡This included any health professional (eg, physiotherapist, nurse, chiropodist) visiting the person in their own home.

§An organised centre where people attend to meet others, socialise and take part in activities.

### Feasibility outcomes

#### Eligibility, recruitment and attrition

Just under half of those assessed met the eligibility criteria (50/106). Although the original recruitment target of 50 was not met, the consent rate was 60% of those eligible. The addition of the new occupational therapists was a potential threat to feasibility insofar as it threatened the control group which did not have routine occupational therapy input and meant that recruitment had to be curtailed, which meant that fewer users could be screened for eligibility. This meant that the recruitment target of 50 was not reached. However, once recruited, participants were willing to remain in the study, and 92% of surviving participants were followed up at the final time point.

#### Suitability of outcome measures

All of the outcome measures showed a change from baseline to 2 weeks in the sample as a whole indicating that these were responsive to change in this group of people. Therefore, the measures appeared appropriate and relevant to the study population. Furthermore, there were some differences between the groups, particularly at the 2-week follow-up, which suggests that these measures have the potential to show a difference between groups, if such a difference exists. With regard to completeness of data collection, 98% of the measures were completed in full, meaning that missing data were minimal and within acceptable limits. However, at the 2-week follow-up, four participants had reached the ‘ceiling’ maximum score of 20 on the BI, indicating that they were fully independent with those ADL. This meant that they had no further potential for improvement on this measure.

#### Content, scheduling and acceptability of the occupational therapy intervention

On the whole, it was possible to schedule treatment visits and deliver the intervention in a way that was consistent with the protocol. The median length of the reablement episode, and therefore the intervention, was 56 days, range 20–126 days. Thus, the reablement episode often lasted longer than the 6-week target; this was primarily due to delays in handing over an ongoing care package to a care agency. The median number of occupational therapy visits per participant was 5, range 2–13. The median occupational therapy visit length was 45 min, range 15–90 min. Overall, each participant received an average of 10 hours of occupational therapy time: 4 hours of direct contact, 3 hours of administration and liaison, and 3 hours of travel time. A total of 28 goals were set for the 13 participants who received the intervention, median 2, range 1–4. All participants were able to set one or more ADL-related goals. The most common areas for goals were bathing/showering (n=8), kitchen activities (n=6), strip washing (n=4) and outdoor mobility (n=3). The occupational therapist's time spent on the particular components of the intervention was recorded on an electronic pro-forma after each visit. The majority of time was spent on assessment (29%), followed by case management and advice and support (24%), practising activities (19%), goal reviewing (12%), teaching techniques (11%) and goal setting (5%).

Acceptability was evaluated using a questionnaire which was sent to all participants in the intervention group and semistructured interviews with five participants in the intervention group. The questionnaire and interviews revealed a high level of satisfaction with the intervention, and participants reported that they believed the intervention helped them to increase their ability to manage ADL.

#### Feasibility of the cost and resource use data collection

It was possible to collect the required data on the time and costs of delivering the intervention. This was recorded on a visit pro-forma which was completed electronically after each treatment visit to participants in the intervention group. Participants were also able to report whether or not they had used health and community services; however, there was some missing data (8%) for the duration of contact with health professionals and services, suggesting that participants were not always able to reliably recall this information.

## Discussion

It was feasible to conduct an RCT of this intervention in this setting. However, there were aspects that worked well and those that were problematic. The main problem was that the recruitment target was not reached principally due to a change in ‘usual care’ at the trial site, which involved the addition of new occupational therapists into the service. This is consistent with the national picture and the trend for increased numbers of therapists in reablement services.^15^ Nevertheless, the eligibility and consent rates were appropriate and were comparable with other rehabilitation trials that were conducted as pilot studies and developed to be funded as full RCTs (eg, the occupational therapy in care homes study^30^ ^31^). Furthermore, participant attrition, for reasons other than death, was extremely low. Overall, it was possible to recruit participants, deliver the intervention as planned, retain participants in the study and collect complete outcome, cost and resource use data.

The principal strength of this study is that it was the first to use a randomised method to evaluate a component of homecare reablement in the UK. There have been few RCTs within UK social care settings to date,^32^ and this study demonstrates the potential for further RCTs in this area. It has also generated data to inform a further RCT of occupational therapy in reablement. However, the study was conducted at a single site, involving only one occupational therapist delivering the intervention, and therefore the findings should not necessarily be considered generalisable to other local authority settings, although there is no obvious reason why they would not be.

As the first feasibility RCT of occupational therapy in reablement, there are no directly relevant studies for comparison. The findings are not definitive and should be interpreted cautiously by clinicians and policymakers. However, the favourable trends for the OT group are consistent with the findings from a systematic review which was completed as a precursor to this study, which showed small, non-statistically significant trends towards improvements personal ADL ability following interventions in homecare, including those involving occupational therapists.^20^ Furthermore, systematic reviews of occupational therapy interventions in other contexts have shown improvements in ability to manage ADL, for example for older adults in the community^33^ and after stroke.^34^ ^35^ It is also important to note that the control group also showed improvement from baseline on several measures, although to a lesser degree than in the OT intervention group overall. Such change is consistent with similar studies showing improvements following reablement as an alternative to traditional homecare, although this change may also be due to natural recovery. Nevertheless, there is still an outstanding and important question in relation to the success of different models of reablement, including directly delivered occupational therapy interventions, and further research is required.

The main implication from this study is that a further powered trial would be feasible. However, proceeding with an RCT analogous with the design of this study would be subject to two important caveats:

1. To identify a sufficient number and range of sites providing reablement *without* routine occupational therapy input in order to establish a control group comparator. However, this may be problematic given the changing local authority landscape. Alternatively, other designs could be considered, such as standard care involving occupational therapists working in other models of service delivery^19^ in comparison to the enhanced programme described here. However, a clear picture of the nature and extent of occupational therapy input into these services would be needed.
2. The main focus of the intervention was ADL within the home; thus, we suggest that a measure of personal ADL is the most appropriate primary outcome. Although we used the BI, 4 participants scored the maximum of 20 at the 2-week follow-up, meaning that they reached the ‘ceiling’ of the scale; this effect is well documented in the literature.^36^ There is therefore scope for further research to identify or develop a more suitable outcome measure for use in homecare reablement. The National Audit for Intermediate Care has also previously reported ‘much debate’ when agreeing which outcome measure to use for home-based intermediate care and reablement services.^15^ While quality of life, physical functioning and mental well-being are all important secondary outcomes, ability to manage ADL within the home is an essential outcome for this service user group.

Given that government policy in the UK is focused on providing reablement services to assist people to remain independently in their homes, the implications of a definitive study in this area are likely to be important. This trial has showed that such a trial is feasible and warranted.
